# Supplementary material for: Mineral dissolution and reprecipitation mediated by an amorphous phase
Source: Nat Commun. 2018 Apr 24;9:1637. doi: 10.1038/s41467-018-03944-z (PMC5915427; doi:10.1038/s41467-018-03944-z)
Supplement: Supplementary file 1 — Supplementary Information [file 41467_2018_3944_MOESM1_ESM.pdf]

Supplementary material

Mineral dissolution and reprecipitation mediated by an amorphous phase

Matthias Konrad-Schmolke et al.

Supplementary Note 1

The samples investigated in this work stem from the late Mesozoic Franciscan Mélange outcropping at the West Coast of California in the United States. The samples were collected at the mouth of the Russian River about 1.5 km west of the town of Jenner, which is about 30 km west of Santa Rosa and about 90 km northwest of San Francisco, CA. Sample coordinates are 38° 27.197'N and 123° 7.941'W. Here the Central Belt mélange unit of the Franciscan Complex is exposed (Supplementary Figure 1). The Franciscan Mélange displays an ancient subduction complex where large parts of the accretionary wedge are exhumed. Within a predominantly fine-grained greenschist to blueschist-facies matrix medium to coarse-grained mafic blocks with varying sizes ranging from cm to decameter can be found. These so-called knockers are exotic blocks in a dominantly argillitic and subordinately serpentinite matrix[1 2 3]. Like most of these knockers the sampled block is a scraped-off part from the subducting plate incorporated into a mélange of rocks at the slab-mantle interface. In this mélange mafic material from the oceanic crust is mechanically mixed with ultramafic material from the overlying mantle and chemically modified by fluids derived from the dehydrating slab (Supplementary Figure 1).

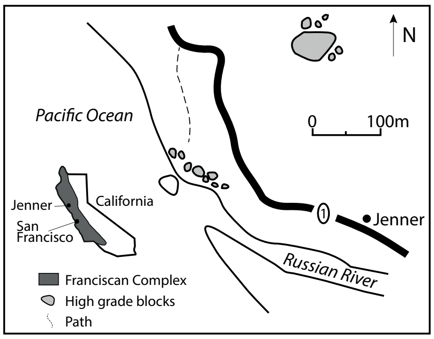

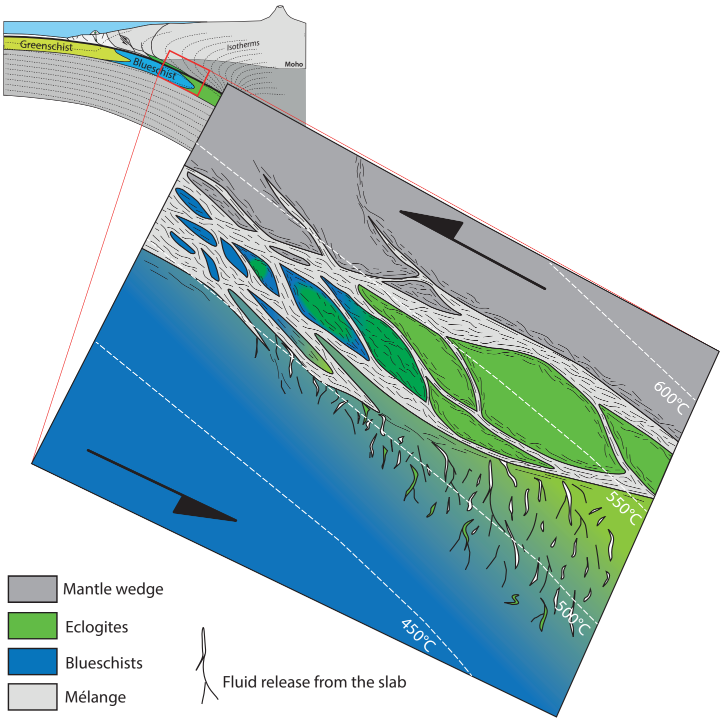

Supplementary Figure 1: Simple sketch maps of the Jenner locality and illustration of the mélange formation. Maps of the Jenner locality (above) redrawn from Krogh et al., 1994[4]. The subducting slab is undergoing dehydration and transformation from blueschist to eclogite facies thereby liberating fluids that migrate into the slab-mantle interface region (left). Eclogitic blocks mechanically scraped off the subducting plate are infiltrated by the liberated fluids and re-hydrated to blueschists.

Several models have been proposed to explain the occurrence of these high-grade blocks in the mélange[4], including transport via buoyant serpentinite diapirs[5 6], specific flow patterns in an accretionary prism[7] and by listric normal faulting at the base of the mantle wedge[8 9]. Today the Franciscan Mélange is one of the best examples of high pressure rocks exhumed along the slab-mantle interface in subduction zones. A model for such an exhumation mechanism has been proposed by Gerya et al. (2002)[10] and the pressure-temperature paths resulting from these models coincide well with those proposed for the Franciscan Mélange and therefore our samples (Supplementary Figure 2). Hence, it is notable that the investigated rocks display one of the best examples of fluid-rock interaction processes in subduction zones, which emphasizes the importance of our findings regarding geodynamic and geochemical processes.

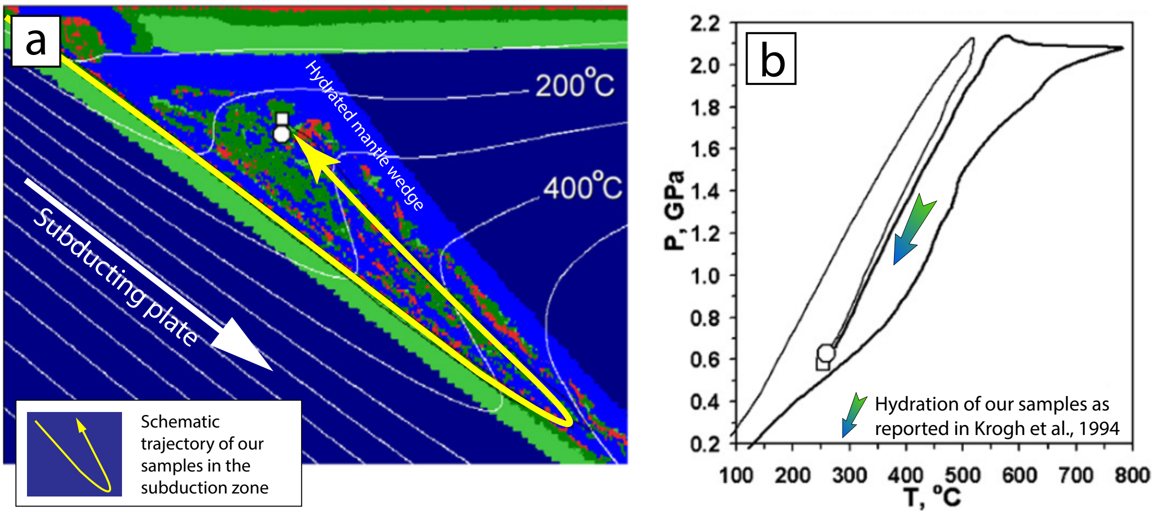

Supplementary Figure 2: Thermomechanical model of high-pressure rock exhumation along the slab-mantle interface. a) The yellow arrow displays the trajectory along which fragments of subducted oceanic crust can be exhumed in a serpentinite-rich hydrated mantle wedge. b) Especially the retrograde part of the modeled P-T path corresponds well with the proposed data in Krogh et al., 1994. The green-to-blue arrow indicates the conditions during which the re-hydration of our samples occurred. Redrawn from Gerya et al., 2002.

The high-grade blocks of the Franciscan have been the subject of numerous geochemical studies which concluded that interaction of the blocks with surrounding ultramafic material occurred based on the presence of high Mg, Ni and Cr contents in metasomatic rinds[2], but geochemical evidence, such as enrichment in large-ion lithophile elements, low εNd and δ^7Li values in blocks and rinds, for interaction with sediments or sediment-derived fluids is also widespread[11 12]. The blocks reached peak metamorphic conditions in the eclogite facies with temperatures of up to 520 °C[3] at around 158 Ma[13], followed by a retrograde blueschist facies overprint at 300-350 °C[14].

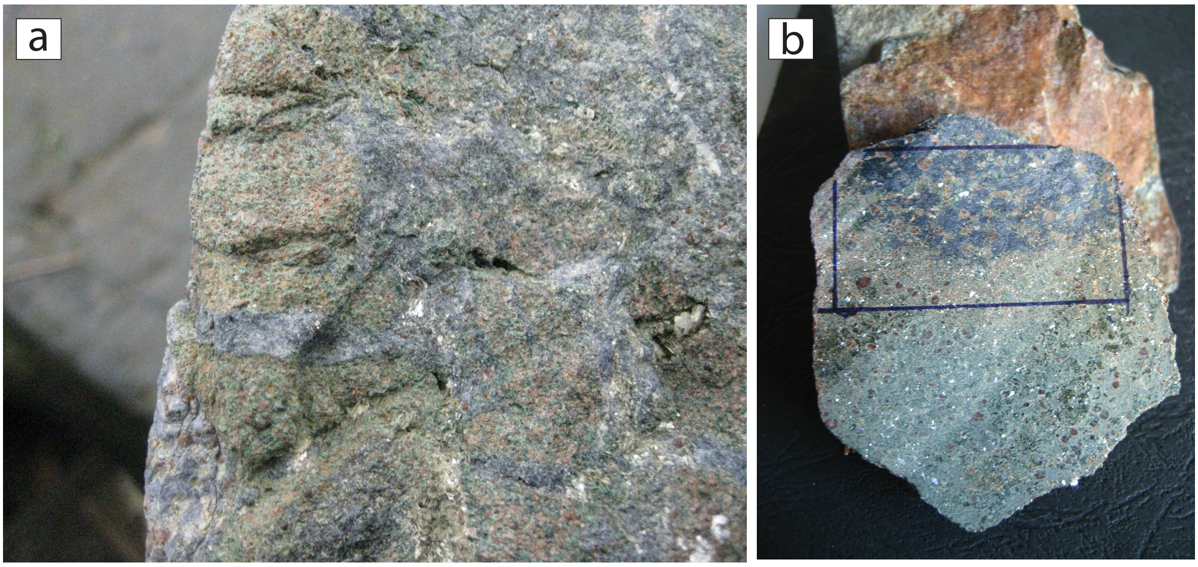

Supplementary Figure 3: Details of the sampled rock. (a) Fluid infiltration into the dry eclogite facies rocks leads to a hydration of the primary assemblage clinopyroxene +

garnet (dark green and red) to form sodic amphibole (dark blue) and to a minor extent white mica (white spots). The fluid influx into the eclogites occurs along discrete fluid pathways (left side of the block), but also pervasive (upper part). Width of the photograph is about 15cm. (b) The infiltration front is irregular, but sharp down to mm scale and the primary texture, such as the porphyroblastic garnet grains in the sample above, are often preserved after the fluid influx. Width of the blue rectangle is 4.5cm.

Supplementary Note 2

The rounded block consists of two parts, a greenish upper part and a dark blue lower part. The color differences clearly reflect the contrasting mineral assemblages in the two parts. The upper greenish part displays a more or less unmodified eclogite-facies mineral assemblage clinopyroxene (cpx) + garnet (grt) + rutile (rt) ± epidote (ep). Sodic calcic amphibole (NaCa-amph) and white mica are less abundant but can be found as inclusions in garnet and pyroxene as well as sporadically in the matrix. This assemblage corresponds to the peak pressure stage 4 of Krogh et al., 1994 (1.5 GPa/550°C). The bluish parts represent those parts of the block that are affected by rehydration, which is indicated by the transformation of cpx and Na-Ca-amph into sodic amphibole (Na-amph), the breakdown of epidote and the formation of titanite from primary rutile. The reaction front between the two parts is in most cases sharp (down to mm-scale, as can be seen in the back scattered electron images) and the primary texture is often preserved after the metasomatic reaction (Fig. 3), which indicates a fluid influx under static conditions. The mineral assemblage in the blueschist-facies part corresponds to the stages 5 and 6 of Krogh et al. 1994 (1.3 GPa/500°C to 1.1 GPa/450°C) and indicates decompression together with cooling with respect to the (preserved, unaffected) eclogitic upper part of the block. The reason for the sharp boundary between the two parts and the preservation of the eclogite-facies assemblage is likely a fluid influx that reached the lower part of the block, but did not reach further into the upper part. This enabled us to study the processes at the reaction- and fluid infiltration front.

The eclogitic parts have a medium- to coarse grained (1-10mm) porphyroblastic texture with a matrix consisting predominantly of medium grained subhedral clinopyroxene (cpx) and coarse grained porphyroblastic euhedral garnet (grt) grains. Finer grained sodic-calcic amphibole (NaCa-amph), white mica (wm) and epidote (ep) occur intersticially in the matrix, but predominantly as inclusions in cpx and grt and are less abundant. Rutile (rt) occurs as an accessory phase predominantly included in cpx and grt but also less abundant in the matrix. The blueschist areas are finer grained (average grainsize ~1mm) and consist predominantly of euhedral sodic amphibole (Na-amph), rare interstitial lawsonite, white mica and titanite that is often growing along the grain boundaries. An important textural difference between the eclogitic and the blueschist areas is the nature of the grain boundaries. These form an open, often interconnected network in the eclogites, but are predominantly closed in the blueschist part of the rock.

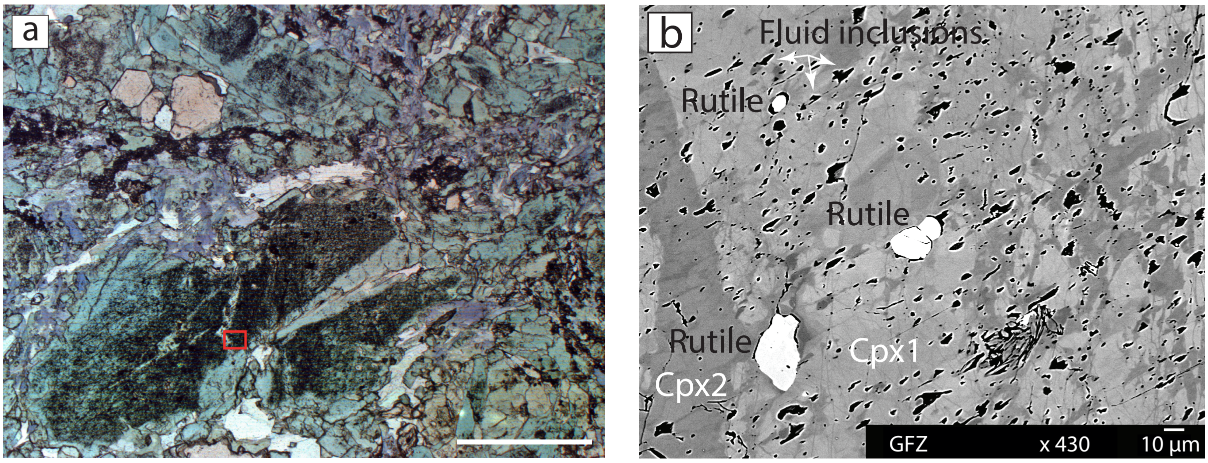

Supplementary Figure 4: Details of the microtextures in the investigated samples. (a) Thin section photograph of an eclogitic area that is weakly affected by fluid influx. The large cpx grains have inclusion-rich cores (dark) and inclusion-poor overgrowths (light green). The inclusions consist of rutile grains and primary fluid inclusions. Secondary sodic amphibole grains (bluish) partly replace the primary cpx. Scale bar is 1mm. (b) Back scattered electron from the red rectangle shown in the left image. The core of the cpx grain (light grey) contains few rutile (white) and abundant fluid inclusions (dark spots). The core (cpx1) is truncated by a network of brittle fractures that are filled with cpx material (cpx2) that is also found as overgrowth along the rims of the primary cpx.

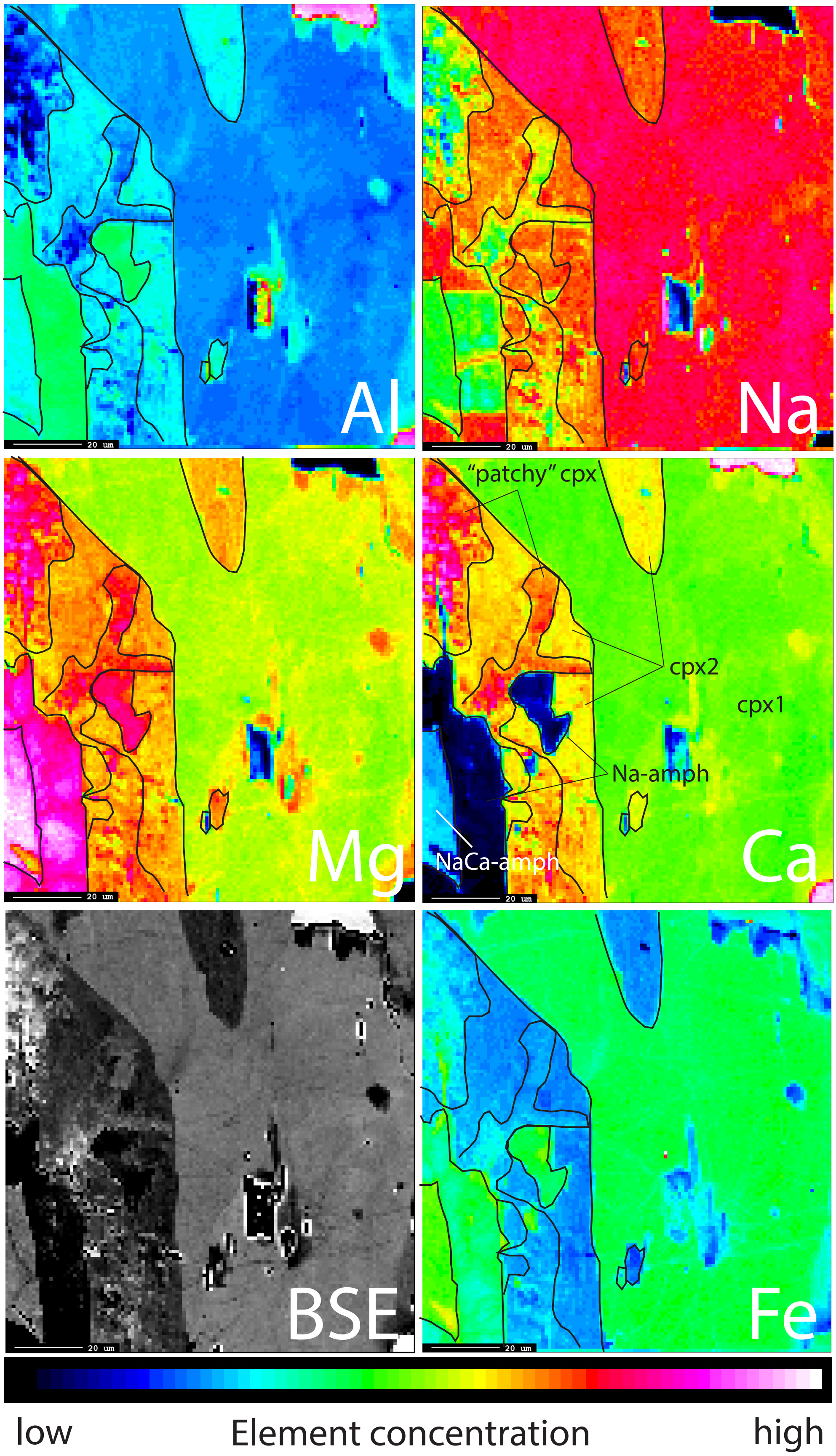

Supplementary Figure 5: Electron microprobe X-ray mapping of the reaction zone between cpx, NaCa-amphibole and Na-amphibole. The area is shown in Fig. 1b of the main text. Four different phases are clearly distinguishable by their characteristic compositions. (1) The large cpx1 crystal covering the right side of the mapping is overgrown by cpx2, which has higher Ca and Mg and lower in Na and Fe concentrations. The sodic-calcic amphibole NaCa-amph) grain on the middle left side can be distinguished by its high Mg content. Sodic amphibole (Na-amph) consuming sodic-calcic amphibole as well as cpx has the lowest Ca

concentrations. The patchy areas in the cpx resulting from the fluid influx have the highest Ca and Mg and lowest Fe and Na concentrations of all cpx generations.

Supplementary Note 3

In general two generations of cpx can be found in the eclogitic part of the block. The first generation forms the cores of the up to several mm large cpx grains. This cpx generation is inclusion-rich (cpx1; Supplementary Figure 4a), where inclusions consist predominantly of fluid inclusions (FI) and rutile (rt) (Supplementary Figure 4b). Cpx1 grains are affected by brittle deformation indicated by a network of cracks that connect many of the FIs and are filled with secondary cpx (cpx2). Cpx2 is also formed along the rims of cpx1 and is slightly lower in Na and Fe and higher in Ca and Mg, indicating growth during decreasing pressure and possibly increasing temperature (Supplementary Figure 5). Such a pressure-temperature evolution is consistent with an incorporation of blocks scraped off from the cooler oceanic plate into the hotter slab-mantle interface region, but is not in full agreement with the anticlockwise P-T trajectory proposed by Krogh et al., 1994. However, detailed geothermobarometry and a related discussion is beyond the scope of this paper.

Cpx2 is further characterized by many porous areas (Supplementary Figure 6) where the pores are between 0.5 and 5 μm wide and often form an interconnected network, which we verified utilizing focused ion beam sections through porous areas (Supplementary Figure 7). The extent of the interconnection of the pores could, however, only be demonstrated in the dimensions of the FIB sections, which are in the order of 50 x 50 x 50μm. Porous cpx2 areas occur along grain boundaries (arrow 1 in Supplementary Figure 6a) as overgrowths on cpx1 grains as well as within larger cpx1 grains (arrows 2 and 3 in Supplementary Figure 6a) where cpx2 can be shown to be the result of epidote breakdown (Supplementary Figure 6b). As evident from Supplementary Figure 6b epidote-derived cpx is highly porous (large arrow in Supplementary Figure 6b) resulting from a negative volume change (~3%) regarding the solid part of this dehydration reaction. Nevertheless, there are also porous areas within the cpx grains that cannot be clearly attributed to a certain mineral reaction (arrow 3 in Supplementary Figure 6a and small arrow in Supplementary Figure 6b).

Supplementary Note 4

There are several characteristic textural and compositional features associated with the subsequent hydration event. Around initial fluid pathways, such as grain boundaries and around porous cpx2 areas clinopyroxene grains develop cloudy and patchy compositional variations (Supplementary Figure 6). The affected areas within the grains are often clearly separated from the unaffected parts by reaction fronts that emerge from the interconnected pore network and penetrate into the grains. The affected areas within the cpx have significantly lower Na and Al and higher Ca and Mg concentrations than all previous cpx generations (Supplementary Figure 5). The bright cloudy parts display areas with the highest Fe and Ca contents and also mark the sites of subsequent nucleation of sodic amphibole (Supplementary Figures 5 and 6c). The fluid induced formation of sodic amphibole is always associated with the occurrence of the cloudy cpx texture and a network of nm- to μm-wide open pores that form reaction fronts penetrating into the existing cpx and sodic-calcic amphibole grains (Fig. 1b-d). The micro- and nano-structures associated with the formation of sodic amphibole are described in the main text.

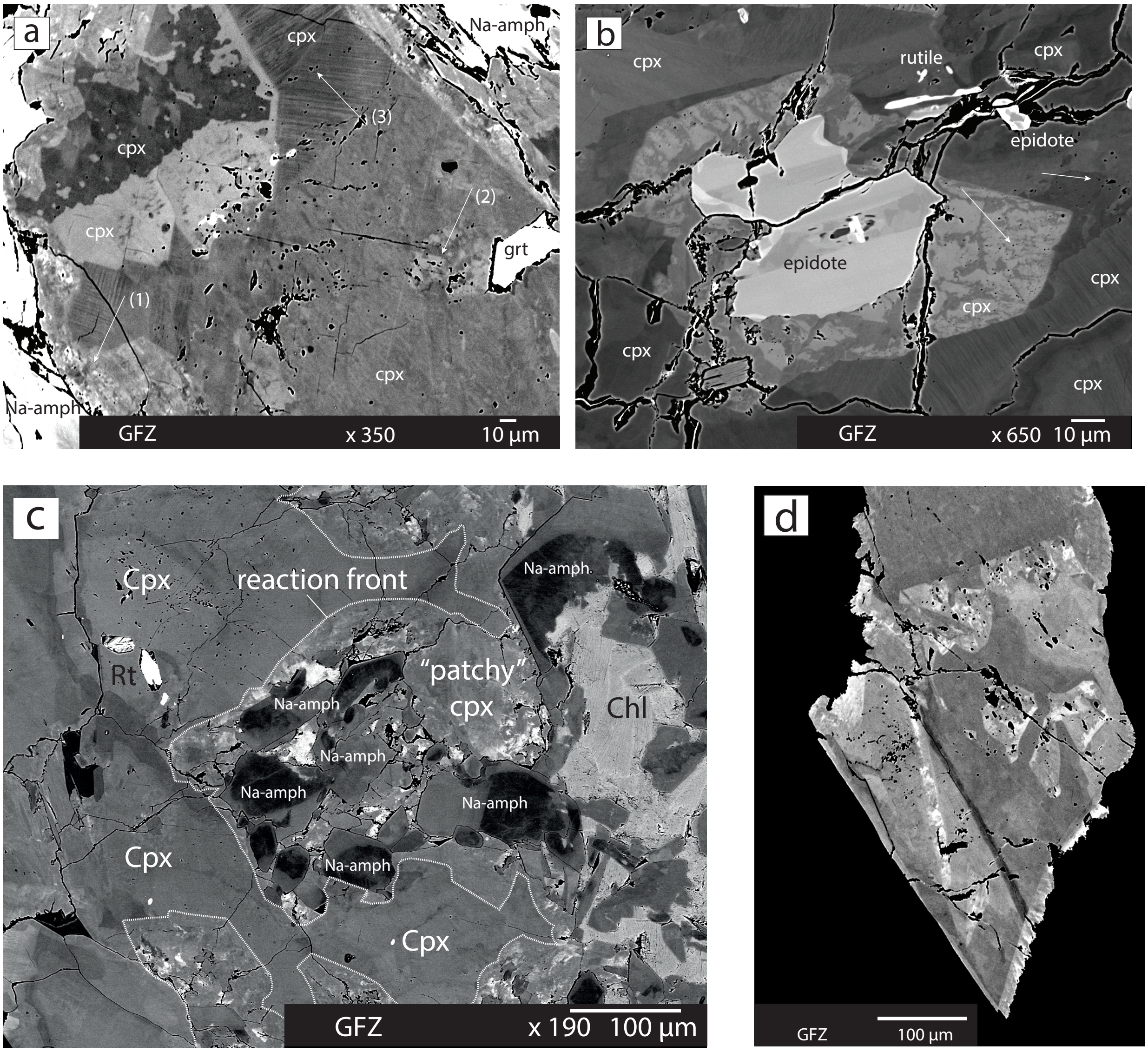

Supplementary Figure 6: Back scattered electron images of porous cpx and fluid influx textures. (a) Porous cpx occurs along the grain boundaries as overgrowth of cpx2 on cpx1 (arrow1), but also within larger cpx1 grains (arrows 2 and 3). In most cases these porous areas are surrounded by patchy zoned cpx (arrows 1 and 2). (b) Porous cpx can result from the breakdown of hydrous epidote, a reaction that is associated with a ~3% negative volume change in the solids. (c) The reaction front is highlighted by the distribution of the patchy zoning within the cpx grains. Subsequent to this cpx modification the formation of sodic amphibole starts in these patchy areas. (d) Characteristic modification of grain boundaries and porous areas as a result of the fluid influx. Most, but not all grain boundaries and porous areas show the bright and patchy zoning.

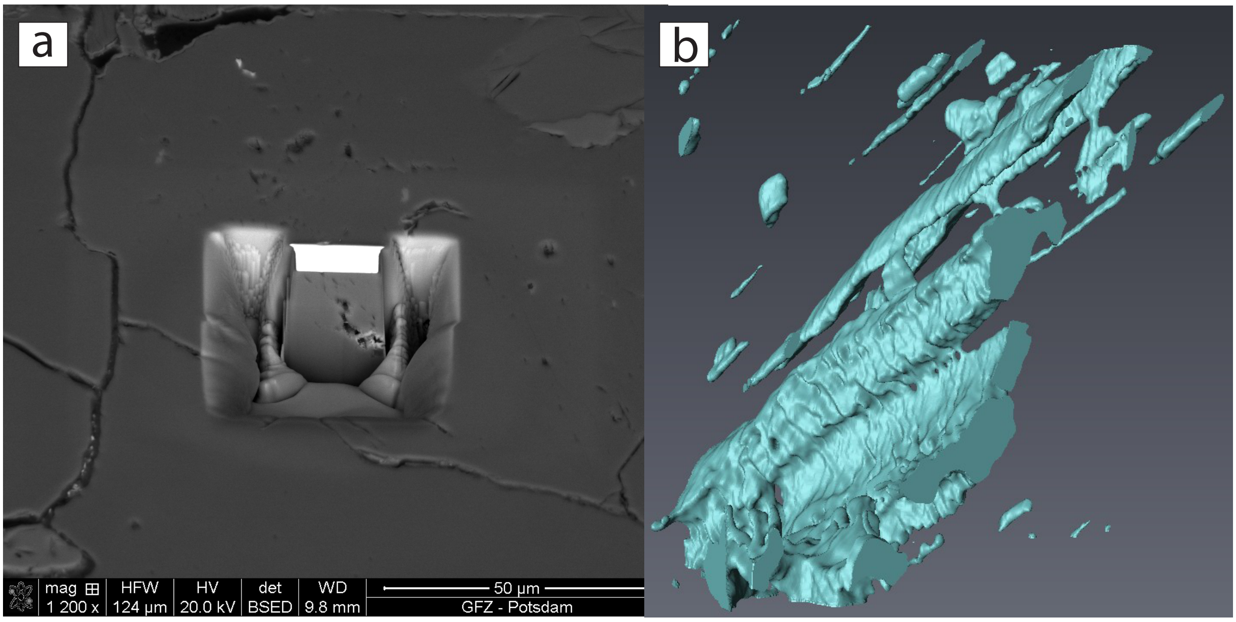

Supplementary Figure 7: Documentation of the microporosity. (a) Back scattered electron image of a focused ion beam pit that was used to determine the extent of the pores into the grains interior. The white rectangle is the remainig Pt coat that ensures conduction. (b) Three-dimensional visualization of the interconnected porosity. The elongated pores are extending across the sampled volume and are interconnected allowing for effective fluid transport within the porous grains. Width of the image is 30µm.

Supplementary Note 5

We sampled the reaction zones between primary cpx and the porous and patchy cpx as well as the boundary between reacting sodic-calcic amphibole and sodic amphibole using a focused ion beam (FIB), in order to produce electron transparent slices, which were then investigated by transmission electron microscopy (TEM). Sample areas are indicated in Figs. 1 b and c. The bright field TEM image of the foil taken across the cpx-cpx reaction zone (Supplementary Figure 8a) shows the reacting cpx on the right side of the image, the newly formed cpx on the left and an Na-amphibole crystal forming in the upper left corner of the foil. The lighter areas in the reacting cpx are defects that are aligned parallel to the 001 direction and define the (110) cleavage plane. The contact zone between the minerals is marked by an elongated pore space that is between 100 nm and 1µm in width. In the middle part of the cpx-cpx contact this pore space is missing and both minerals are in direct contact (Supplementary Figure 8a). These textural relations suggest that the replacement of cpx1 occurs in an interface-coupled dissolution re-precipitation reaction.

The reacting surface of the cpx1 undergoing dissolution is highly irregular and decorated with micro-denticles that indicate preferred consumption of the reacting cpx in the 001 crystallographic direction. As shown in the main text these denticles grade into a mesh of nm sized needles that ultimately form an amorphous material that is in contact with the newly formed Na-amphibole.

The surface of the newly-formed cpx is in contrast more smooth with only a few humps in the lower part of the pore space (Fig. 2a). Denticles on cpx3 develop only in the vicinity of the precipitating Na-amphibole where it is finally consumed. Both observations

indicate that the reaction textures as well as the pore space are syn-metamorphic features. This needle-like texture as well as the amorphous phase associated with it can be observed in different contexts within our samples. Supplementary Figure 8b shows a close up BSE image of one of the cpx grains formed from epidote. The cpx formed from epidote is highly porous and has at the upper edges a denticulated contact with cpx1. A pore space as in Supplementary Figure 8a is missing. This lack of a porous region between the grains either suggests that an existing pore space was only transient and that cpx material recrystallized and filled the pores or that the entire re-crystallization from cpx1 to cpx2 occurred along dislocations, such as those in Supplementary Figure 8a, not involving any (visible) pores at all.

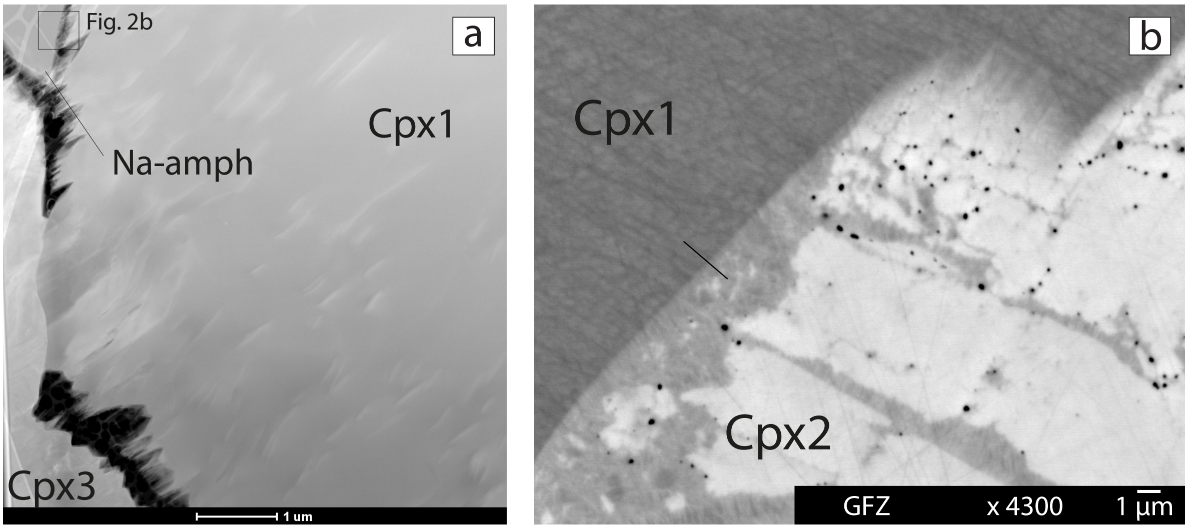

Supplementary Figure 8: Details of the cpx decomposition and chemical modification along dislocations. (a) Dark field TEM image of the FIB section shown in Fig. 3. The cpx1 crystal on the right side has plenty of dislocations visible as lighter areas as well as a denticulated surface along the entire grain boundary. The dislocation-rich areas correspond to the patchy zoning in cpx1. The newly formed cpx3 on the left side has a straight grain boundary with cpx1, but a denticulated surface where it is in contact with the sodic amphibole. These observations suggest formation of cpx3 from cpx1 and subsequent consumption of cpx3 during the formation of sodic amphibole. Note also the contact between cpx1 and cpx3 indication an interface-coupled dissolution reprecipitation mechanism during the reaction. (b) BSE image of the contact between a more or less unzoned cpx1 crystal and a patchy and porous cpx grain that was formed from epidote. The black line indicates the position of the TEM foil shown in Supplementary Figure 9.

The amorphous material occurs also as precipitates on the denticulated cpx surface (Supplementary Figure 9). The overview of the FIB section taken across a pore associated with the formation of gln shows a several µm large pore that is in the internal surface decorated with a darker amorphous material. The white material in the pore interior is precipitated Ga from the FIB sectioning. The close up bright field TEM image shows that the amorphous material precipitated on the denticulated cpx surface and has several worm-like appendices reaching into the pore interior. The denticles of the dissolving cpx are grading into a fine mesh and seem to be dissolved into the amorphous material. This is visualizing the dissolution process described in the main text and shown in the HR TEM images in Fig. 3b.

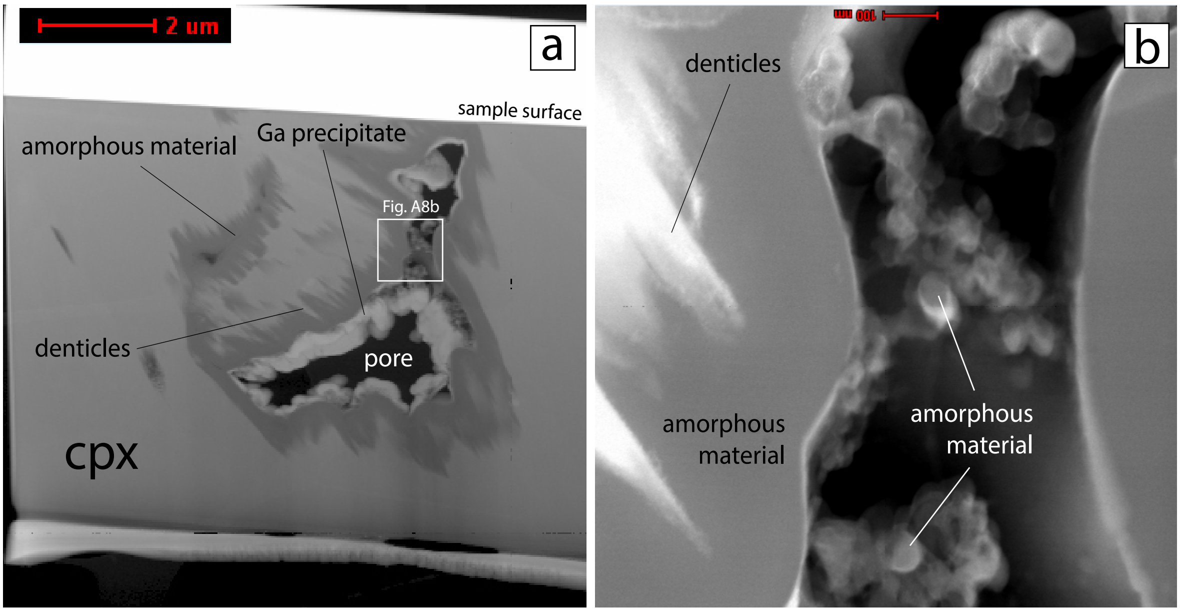

Supplementary Figure 9: FIB section across a porous reaction zone with inner wall coatings of amorphous material. (a) Overview image of a TEM foil taken across a pore within cpx. The darker areas display amorphous material that precipitated on the denticulated surface of the reacting cpx. The white precipitates are Ga from the focused ion beam that was used to cut the TEM foil. (b) Close up of the narrow part of the pore showing the denticles grading into amorphous material as well as worm-like appendices reaching into the pore space from the surface of the amorphous material. The precipitation of the amorphous material on the denticulated surface indicates that it is mobile in the pores and that it coexists with the denticles. The dissolving denticles demonstrate the formation of the amorphous phase directly from the solid (cf. Fig. 4). The composition of the amorphous phase is similar to that shown in Fig. 4b.

Supplementary Note 6

Another important finding is the connection between the dislocation density in the mineral grains and the cloudy compositional variations visible in the BSE images. The FIB section shown in Supplementary Figure 10 is taken along the black line indicated in Supplementary Figure 8. The left side of the FIB slice is representing the more or less homogeneous cpx, whereas the right side is sampling the patchy zoned secondary cpx. The distribution of the dislocations, visible as light gray areas in the FIB slice, correlates well with the visible compositional variations in the BSE image (cf. Supplementary Figure 8b and Supplementary Figure 10). The homogeneous part on the left side has a low dislocation density, whereas in the patchy part dislocations are abundant (Supplementary Figure 8b). This unambiguous correlation indicates a connection between the dislocations and the compositional variations in the mineral grains. We interpret this correlation in a way that the dislocations serve as fluid pathways along which element exchange is enabled after the crystallization of the crystal. The compositional pattern visible in the BSE image is not a primary feature, but reflects differences in the dislocation density among the different parts of the mineral. These differences became visible after the fluid influx modified the crystals along the dislocation lines. The cloudy areas around the porous regions, the fluid infiltration fronts along the grain boundaries as well as the patchy zoning in the cpx replacing epidote are all the result of the fluid infiltration, which facilitates the interpretation of the extremely complex zoning pattern in the cpx grain in Fig. A6a. These observations underline that fluid migration and element exchange

pathways can be as small as several nm and, if these dislocation-rich areas within the crystals can be generated by the differential stress exerted by the fluid on the solids, pervasive fluid migration is not restricted to the grain boundary network or syn-metamorphic porosity, but can create its own pathways even through mineral grains.

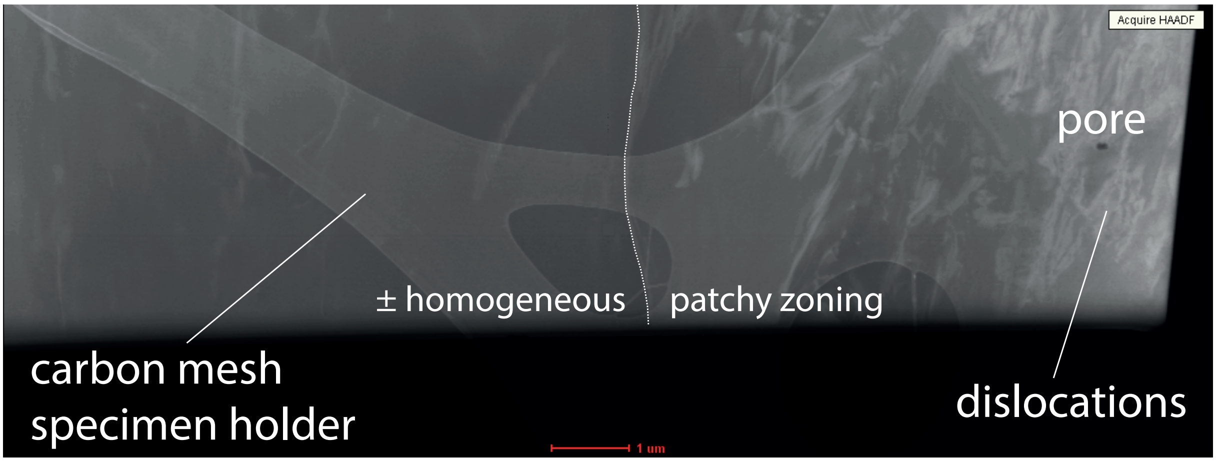

Supplementary Figure 10: Overview of a TEM foil taken across two cpx grains with different dislocation densities. The FIB section is taken along the profile shown in Supplementary Figure 8. There is an unambiguous correlation between the dislocation-rich (light gray) areas in the TEM foil and the compositional variations in the BSE image (Supplementary Figure 8b). This correlation indicates that the compositional modification of the patchy zoned grain occurred along the dislocations demonstrating that fluid and element exchange pathways can be as small as several nanometers and reach well into the reacting crystals.

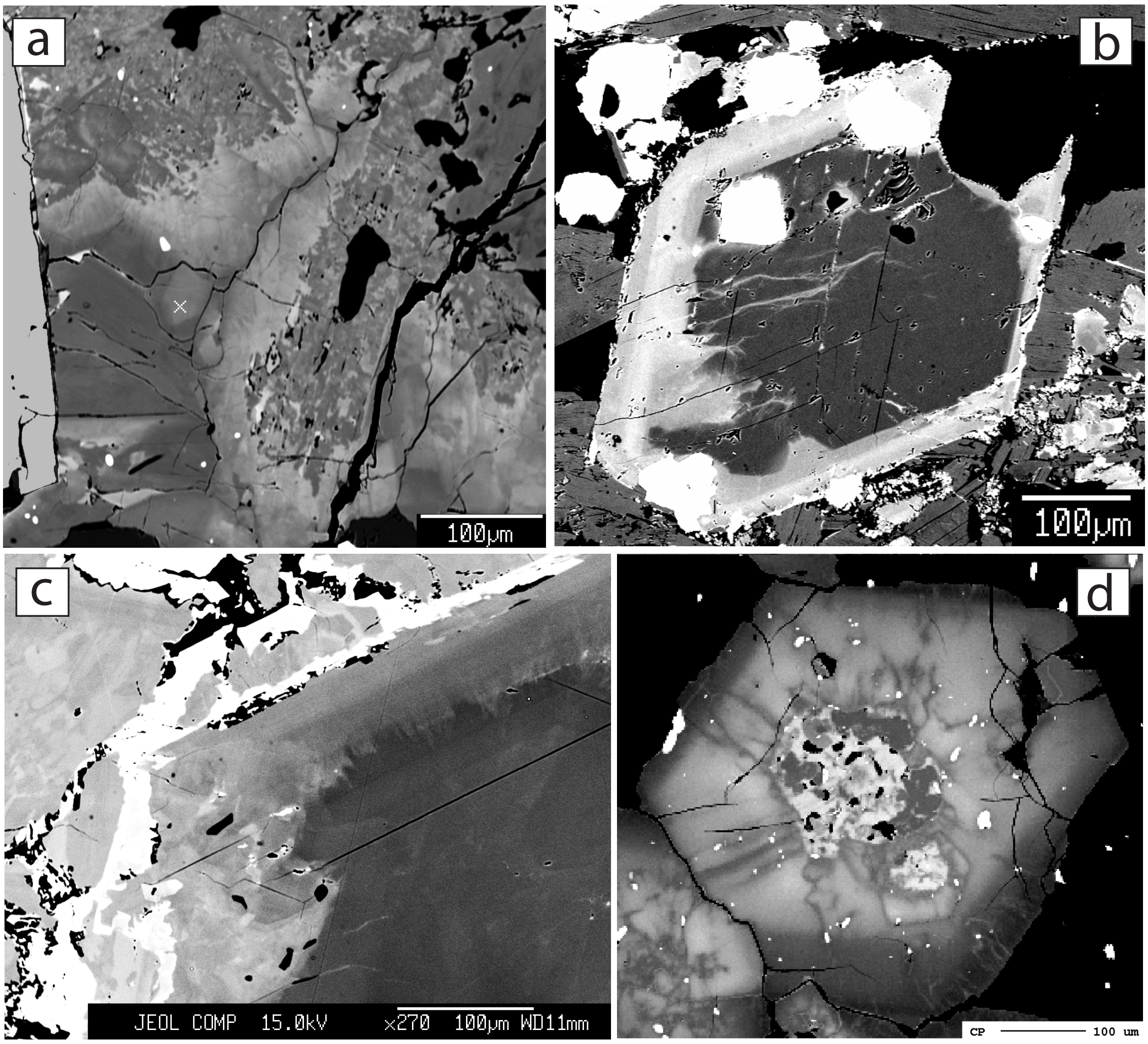

Supplementary Figure 11: BSE images of metasomatic textures in different minerals from different locations indicating the same transformation mechanism as shown in our samples. (a) Cpx crystals from Lago di Cignana ultra-high pressure rocks with patchy zoning and finger-like modifications reaching into the grain interior. (b) Sodic amphibole crystal from the high pressure Sesia-Lanzo zone in the Western Alps[15]. The metasomatic overprint is along grain boundaries, but reaches well into the grains. (c) Sodic amphibole grain from the ultra-high pressure locality at Tso Morari, India. Also here the compositional modifications occur along finger-like structures that enable element exchange with the grain interior. (d) Garnet grain from the ultra-high pressure rocks at Verpennesset, Western Gneiss Region, Norway. Element exchange occurs along channels that connect the garnet interior with the grain boundaries. These textures have been described as element exchange along sub-grain boundaries[16]. Note the formation of new phases in the garnet cores causing an often-observed atoll garnet texture. These examples demonstrate the extensive importance of the findings in this paper.

It is important to note that the here presented compositional features in minerals are not a singularity, but rather often occurring features in high pressure/low temperature rock as demonstrated in the examples shown in Supplementary Figure 11.

Supplementary Table 1: Electron Probe Microanalyzer measurements of the TEM foils

Pyroxene in TEM foil

wt%	**Al2O3**	**CaO**	**FeO**	**Na2O**	**Cl**	**SiO2**	**MgO**	**H2O**	**Total**
3072_CPx1	2,64	4,22	3,01	2,72	b.d.l.	18,00	2,41	-	33,00
normalized									
3072_CPx1	8,00	12,79	9,12	8,24	0,01	54,54	7,30	-	100,00

Amphibole in TEM foil

wt%	**Al2O3**	**CaO**	**FeO**	**Na2O**	**Cl**	**SiO2**	**MgO**	**H2O**	**Total**
3072_Gln1	2,50	0,13	5,28	2,92	b.d.l.	15,61	2,66	-	29,10
normalized									
3072_Gln1	8,59	0,45	18,14	10,03	b.d.l.	53,64	9,14	-	100,00

Amorphous phase in TEM foil

wt%	**Al2O3**	**CaO**	**FeO**	**Na2O**	**Cl**	**SiO2**	**MgO**	**H2O**	**Total**
3072_hole3zooming	0,63	0,05	2,67	1,02	b.d.l.	3,50	0,75	21,37	8,63
3072_hole4zooming	0,64	1,13	1,24	0,85	b.d.l.	3,91	0,81	21,43	8,57
3072_hole5zooming	0,43	1,33	1,09	0,51	b.d.l.	3,53	0,77	22,34	7,66
average	0,56	0,84	1,67	0,80	b.d.l.	3,65	0,77	21,71	8,29
**cations**	**Al**	**Ca**	**Fe**	**Na**	**Cl**	**Si**	**Mg**	**H**	**Total**
	0,01	0,02	0,02	0,03	-	0,06	0,02	2,31	2,47
mol% cations	0,45	0,61	0,94	1,05	-	2,47	0,77	93,72	6,28
weight of the cations	26,98	40,08	55,85	22,99		28,09	24,31	1,01	
weight cations	12,04	24,39	52,56	24,06	-	69,28	18,84	94,65	295,82
wt % cations	4,07	8,24	17,77	8,13	-	23,42	6,37	32,00	100,00

Calculations of the total dissolved solids in the amorphous phase

Normalizing the anhydrous material to 100% totals

	**Al2O3**	**CaO**	**FeO**	**Na2O**	**Cl**	**SiO2**	**MgO**	**H2O**	**Total**	**TDS**
3072_hole3zooming	2,10	0,18	8,90	3,41	0,02	11,67	2,49	71,24	28,76	
3072_hole4zooming	2,13	3,76	4,12	2,84	b.d.l.	13,03	2,69	71,43	28,57	
3072_hole5zooming	1,42	4,44	3,65	1,71	b.d.l.	11,77	2,56	74,46	25,54	
average	1,88	2,79	5,56	2,65	0,01	12,16	2,58	72,38	27,62	
**cations**	**Al**	**Ca**	**Fe**	**Na**	**Cl**	**Si**	**Mg**	**H**	**Total**	
	0,04	0,05	0,08	0,09	-	0,20	0,06	7,70		
weight of the cations	26,98	40,08	55,85	22,99		28,09	24,31	1,01		

weight of the cations	0,99	2,00	4,32	1,98	-	5,69	1,55	7,78	24,31	
wt% cations	4,07	8,24	17,77	8,13	-	23,42	6,37	32,00	100,00	**68,00**
										sum of the cations (wt%)
The amorphous material occupies about 20%vol of the pore and is assumed to have a density of 1 g/cm^3										
wt% cations	0,81	1,65	3,55	1,63	0,00	4,68	1,27	6,40	20,00	**13,60**
										20% of sum of the cations (wt%)
										in g/kg water **136,00**

Using a Monte Carlo Simulation (Casino V2) the interaction volume of the electrons in pyroxenes was determined to be ~0.7µm^3. The depth of electron penetration was restricted by the thickness of the FIB section (≤150nm), which gave an obtainable volume of 0.17-0.23µm^3 that correspond to 25-33% of the maximum interaction volume of ~0.7µm^3. Since the upper part or layers of the interaction volume always produces more x-rays than the lower ones the measured 30 wt% totals in the anhydrous pyroxenes, reflecting the maximum counts obtainable from the TEM foils, correspond well with the simulated value.

Supplementary References

[1] Bailey, E. H., Irwin, W. P. & Jones, D. L. (1964). The Franciscan and related rocks and their significance in the geology of western California. *California Division of Mines and Geology Bulletin*, **183**, 177 p.

[2] Coleman, R. G., & Lanphere, M. A. (1971). Distribution and age of high-grade blueschists, associated eclogites, and amphibolites from Oregon and California. *Geological Society of America Bulletin*, **82**, 2397–2412.

[3] Krogh, E. J., Oh, C. W., & Liou, J. C. (1994). Polyphase and anticlockwise P-T evolution for Franciscan eclogites and blueschists from Jenner, California, USA. *Journal of Metamorphic Geology*, **12**(2), 121-134.

[4] Wakabayashi, J., Moores, E. M., Sloan, D., & Stout, D. L. (1999). Subduction and the rock record: Concepts developed in the Franciscan Complex, California. Classic Cordilleran concepts: A view from California. *Geological Society of America Special Paper*, **338**, 123-133.

[5] Ernst, W. G. (1970). Tectonic contact between the Franciscan melange and the Great Valley Sequence, crustal expression of a late Mesozoic Benioff Zone: *Journal of Geophysical Research*, **75**, 886–902.

[6] Horodyskyj, U., Lee, C. & Luffi, P. (2009). Geochemical evidence for exhumation of eclogite via serpentinite channels in oceancontinent subduction zones. *Geosphere*, **5**, 426–438

[7] Cloos, M. (1982). Flow melanges: numerical modeling and geologic constraints on their origin in the Franciscan subduction complex, California. *Geological Society of America Bulletin,* **93**, 330–345

[8] Platt, J. P. (1986). Dynamics of orogenic wedges and the uplift of high-pressure metamorphic rocks. *Geological Society of America Bulletin*, **97**, 1037-1053.

[9] Ring, U., & Brandon, M. T. (1994). Kinematic data for the Coast Range fault and implications for exhumation of the Franciscan subduction complex. *Geology*, **22**, 735–738.

[10] Gerya, T. V., Stöckhert, B., & Perchuk, A. L. (2002). Exhumation of high-pressure metamorphic rocks in a subduction channel: A numerical simulation. *Tectonics*, **21**(6).

[11] Sorensen, S. S., Grossman, J. N., & Perfit, M. R. (1997). Phengite-hosted LILE enrichment in eclogite and related rocks: implications for fluid-mediated mass transfer in subduction zones and arc magma genesis. *Journal of Petrology*, **38**(1), 3-34.

[12] Penniston-Dorland, S. C., Sorensen, S. S., Ash, R. D., & Khadke, S. V. (2010). Lithium isotopes as a tracer of fluids in a subduction zone mélange: Franciscan Complex, CA. *Earth and Planetary Science Letters*, **292**(1-2), 181-190.

[13] Anczkiewicz, R., Platt, J. P., Thirlwall, M. F., & Wakabayashi, J. (2004). Franciscan subduction off to a slow start: evidence from high-precision Lu–Hf garnet ages on high grade-blocks. *Earth and Planetary Science Letters*, **225**(1-2), 147-161.

[14] Tsujimori, T., Sisson, V. B., Liou, J. G., Harlow, G. E., & Sorensen, S. S. (2006). Very-low-temperature record of the subduction process: A review of worldwide lawsonite eclogites. *Lithos*, **92**(3-4), 609-624.

[15] Konrad-Schmolke, M., Zack, T. and O'Brien, P.J. & Jacob, D. (2008b). Combined thermodynamic and rare-earth-element modelling of garnet growth during subduction:

Examples from the Western Gneiss Region, Norway. *Earth and Planetary Science Letters*, **272**, 488-498.

[16] Konrad-Schmolke, M., O'Brien, P. J., & Heidelbach, F. (2007). Compositional re-equilibration of garnet: the importance of sub-grain boundaries. *European Journal of Mineralogy*, **19**(4), 431-438.
